# Unusual Case: Ophthalmomyiasis

**DOI:** 10.1590/0037-8682-0110-2020

**Published:** 2020-11-13

**Authors:** Fatma Kesmez Can, Handan Alay, Emine Çinici

**Affiliations:** 1 Atatürk University, Faculty of Medicine, Department of Infectious Diseases and Clinical Microbiology, Erzurum, Turkey.; 2 Ataturk University, Faculty of Medicine, Department of Eye Diseases, Erzurum, Turkey.

**FIGURE 1:**
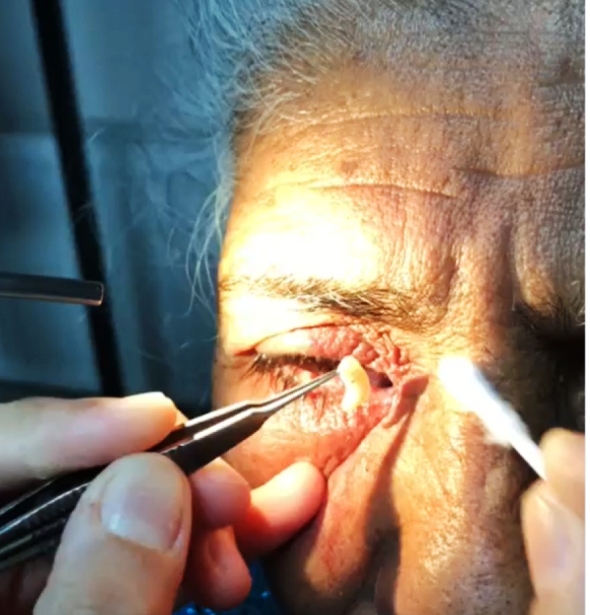
Larva extraction.

**FIGURE 2:**
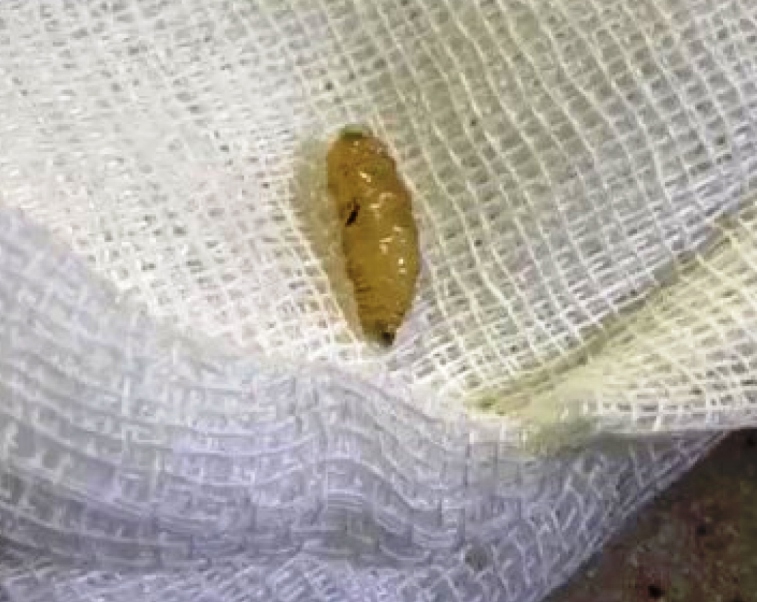
Image of larva.

A 55-year-old woman was admitted to the Eye Clinic at Atatürk University Medical Faculty Hospital in July 2019 due to redness, stinging, and itching in her right eye. At the ophthalmological examination, visual acuity in both eyes was complete and intraocular pressure was normotonic. External examination revealed hyperemia and edema in the right eye, especially on the interior of both eyelids. The left eye was evaluated as normal at the anterior segment examination, while conjunctival hyperemia was present in the right eye, together with a 2-cm long tunnel in the medial canthus with an approximately 0.5-cm wide mouth, extending inwardly and containing larvae. Five moving parasites were detected in this tunnel. The bilateral eyes were normal at the fundus examination. With a local anesthetic drip, the parasites were removed from the right eye using forceps. Irrigation was performed with Batticon and topical antibiotherapy was applied (Figure 1 and Figure 2). The larvae were sent to the microbiology laboratory. Infestation by myase fly larvae was suspected. The patient’s history, symptoms, and findings and the features of the larvae were compatible with ophthalmomyiasis externa^1^. Ampicillin sulbactam (4x1) and local antibiotics were administered for three days. No complications developed and the symptoms regressed. The patient was living in a rural area and worked in animal husbandry. This condition, which is mostly observed in animals, has recently begun appearing in different locations, especially in individuals engaged in agriculture and animal husbandry^2^ ^,^ ^3^. Such individuals should take care and seek ophthalmological advice immediately in case of symptoms.
